# Histoid leprosy presenting as a large tumor^☆^^☆☆^

**DOI:** 10.1016/j.abd.2021.04.009

**Published:** 2021-09-10

**Authors:** Tânia Rita Moreno de Oliveira Fernandes, Victor Josuan Soares de Andrade, Igor Dutra Nascimento, Ana Kívia Silva Matias

**Affiliations:** Department of Medical-Surgical Pathology of the Integumentary Apparatus, Universidade Federal do Vale do São Francisco, Petrolina, PE, Brazil

**Keywords:** Leprosy, Leprosy, lepromatous, Leprosy, multibacillary

## Abstract

Histoid leprosy is a rare form of multibacillary leprosy, characterized by the presence of papules, plaques, or nodules whose appearance is keloid-like, skin colored, or erythematous. Fusiform cells are the main histopathological feature. Due to the fact that it can simulate other dermatological lesions, for example, dermatofibroma and neurofibroma, it constitutes a diagnostic challenge for clinicians and pathologists. It is a bacilliferous form of leprosy, and it plays an important role in disease transmission. A case of a patient with histoid leprosy living in the Northeast Region of Brazil is reported.

## Introduction

Leprosy is a chronic infectious disease whose etiological agent is *Mycobacterium leprae*. Depending on the immunological profile of each individual, the disease manifestations can vary from dermatological macular lesions with altered sensitivity to severe lesions in peripheral, osteoarticular, and vascular nerves, resulting in important sequelae and psychosocial consequences.^1^

In 2018, the World Health Organization reported 208,619 new cases of the disease. Brazil contributed with 92.6% of these cases in the Americas in that same year, while the state of Bahia notified 2,119 new cases, an annual detection coefficient of 14.31/100,000 inhabitants, considered “high endemicity” and above the national average.^2^, ^3^

Histoid leprosy (HL) was initially described by Wade, in 1963, as a rare presentation of drug resistance occurring in multibacillary patients, mainly in lepromatous leprosy (LL), but also in borderline forms, undergoing treatment with dapsone in monotherapy. However, it can appear in patients without previous treatment and, therefore, is not considered a resistance pattern, given its response to traditional polychemotherapy. It accounts for less than 4% of leprosy cases, with male predominance and a mean age between 21 and 40 years. As the bacillary load is very high in these patients, they can become a potential reservoir of infection in the community.^4^

We report an HL patient with exuberant skin manifestations demonstrating its polymorphism and highlighting the relevance of early diagnosis and treatment.

## Case report

**Figure 1:**
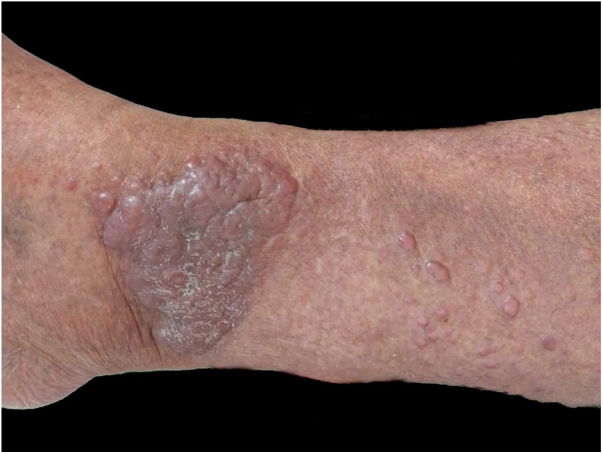
Erythematous, keloid-like papules and large tumor on the ankle.

**Figure 2:**
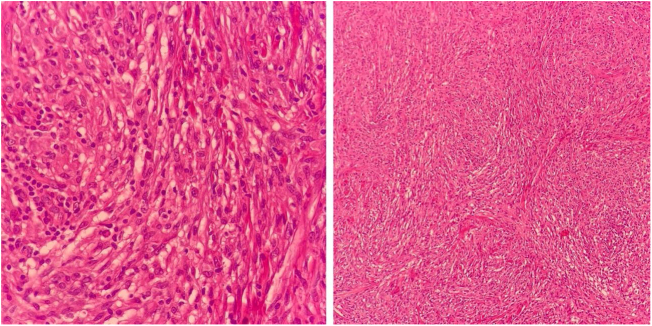
Fusiform histiocytes, arranged in short bundles, occupying the dermis (Hematoxylin & eosin, ×100 and ×400).

**Figure 3:**
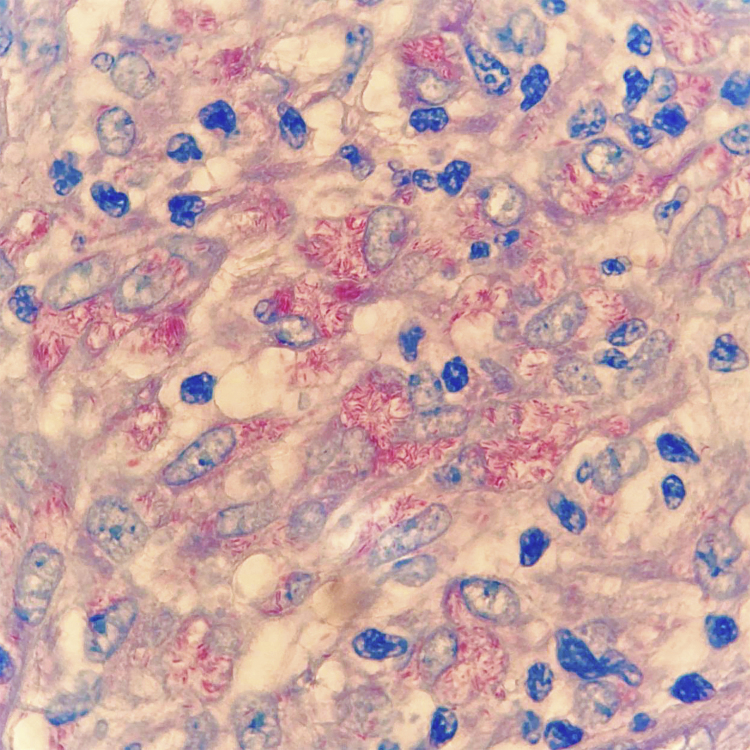
Presence of numerous AFB with Fite-Faraco staining, ×1000.

A 50-year-old male patient, born and residing in the municipality of Sento Sé, state of Bahia, Brazil, was seen at an outpatient clinic reporting the onset of lesions in the trunk, lower and upper limbs approximately 18 months before, with no change in sensitivity or any systemic symptoms. The patient reported having been examined by health professionals, but without a diagnostic conclusion. He reported the use of enalapril, ASA, amlodipine, and spironolactone for the treatment of hypertension. As for his medical history, he mentioned an acute myocardial infarction four years before and treatment for leprosy 10 years before. On dermatological examination, he had papules and nodules, from normochromic to yellowish erythematous, well delimited, with a smooth and shiny surface, which varied between 0.3 and 0.5 cm, with a firm consistency and keloid-like appearance, in addition to a tumor measuring approximately 10/12 cm, indurated, and of a purplish erythematous color, similar to a giant dermatofibroma in the right ankle (Fig. 1). The entire skin was infiltrated, but there was no madarosis or leonine facies. Due to a diagnostic suspicion of LL with a histoid component, a biopsy of the skin tumor lesion was performed for anatomopathological analysis and showed fusiform histiocytes arranged in short bundles, occupying the dermis and the presence of numerous acid-fast bacilli (AFB) with Fite-Faraco staining (Figure 2, Figure 3). A bacilloscopy was also requested, which was positive, with a bacillary index of 1.8 with intact and fragmented bacilli, in addition to normal complete blood count, liver and kidney function tests. Due to the typical clinical picture, he was referred to a basic health unit with a prescription for multibacillary polychemotherapy.

## Discussion

HL is clinically recognized by the presence of keloid-like, normochromic or erythematous papules, plaques or nodules, ranging in size from 1.5 to 3 cm, although much larger lesions have been described, as in the case of the patient in the present report. There may also be involvement of peripheral nerves. Histopathology is crucial for establishing the diagnosis and is characterized by an infiltrate, predominantly constituted by fusiform histiocytes, similar to fibroblasts and which, at times, simulate a fibrohistiocytic neoplasm, associated with few foamy macrophages and numerous acid-fast bacilli. Among its differential diagnoses are dermatofibromas, dermatofibrosarcoma protuberans, xanthomas, keloids, neurofibromas, reticulohistiocytosis, sarcoidosis, and skin metastases. In their most active form, histoid papules and nodules expand rapidly, producing collagen pseudocapsules.^4^, ^5^

Bacilloscopy of histoid lesions shows abundant AFB in clusters, isolated or tightly packed in macrophages. These bacilli appear to be longer with tapered ends when compared to common leprosy bacilli. Its etiopathogenesis is believed to be related to increased cell-mediated immunity, with CD4-lymphocytes, and activated macrophages in the lesions, but with reduced phagocytic properties and humoral production of "suppressor" cytokines, such as interleukin-10 under the influence of bacillary antigens, inhibiting CD4-T cells. These would not adequately present the antigens to the effector cells of the immune system, which could explain the origin of hyperplastic lesions with numerous bacilli and scarce globi formation.^5^, ^6^, ^7^

## Conclusion

Although HL is not the usual form of presentation of multibacillary leprosy and is a diagnostic challenge for clinicians and pathologists, it is important to look closely in endemic areas for atypical forms of leprosy, which, because they are highly bacillary, contribute to maintaining disease transmission.

## Financial support

None declared.

## Authors’ contributions

Tânia Rita Moreno de Oliveira Fernandes: Design and planning of the study; analysis and interpretation of data; approval of the final version.

Victor Josuan Soares de Andrade: Design and planning of the study; analysis and interpretation of data; approval of the final version.

Igor Dutra Nascimento: Design and planning of the study; analysis and interpretation of data; approval of the final version.

Ana Kívia Silva Matias: Design and planning of the study; analysis and interpretation of data; approval of the final version.

## Conflicts of interest

None declared.
